# Assessment and analysis of agricultural non-point source pollution loads in Henan, China: 2001–2023

**DOI:** 10.1371/journal.pone.0336116

**Published:** 2025-12-05

**Authors:** Cangyu Li, Ming Cai, Xinhui Wang

**Affiliations:** 1 Yellow River Engineering Consulting Co., Ltd, Zhengzhou, China; 2 College of Plant Protection, Henan Agricultural University, Zhengzhou, China; Center for Research and Technology Transfer, VIET NAM

## Abstract

Agricultural non-point source pollution (ANPSP) is one of the important factors leading to water environmental pollution. Identifying the spatial distribution of ANPSP and implementing regional control measures are, therefore, important for ensuring effective pollution prevention and control. However, analyzing regional ANPSP using a single approach is challenging due to the impacts of geographical, economic, and policy differences. In this context, the present study aims to assess the long-term spatiotemporal characteristics of pollutants and their sources in Henan Province over the 2001–2023 period using inventory analysis, equal standard pollution load method, and cluster analysis. In addition, we investigated the decoupling relationship between ANPSP and agricultural output value using the Tapio decoupling model. The results showed that: (1) distinct variation stages of total pollution, including total emission reduction, structural transition, and emerging conflicts. Specifically, there was a increase in total pollution over the 2001–2006 period, followed by a fluctuation, continuous decrease, and stabilization in the 2007–2013, 2014–2019, and 2020–2023 periods, respectively. The pollution loads of chemical oxygen demand (COD), total nitrogen (TN), and total phosphorus (TP) were reduced by 26.2, 23.5, and 18.2%, respectively. In addition, increases in the contribution rates of livestock and farmland straw. On the other hand, rural households and livestock were the main sources of COD and TP emissions, respectively. The main source of TN emissions has shifted from livestock to farmland straw; (2) the total pollutant load exhibited a distinct spatial distribution pattern. Specifically, the southern part of the study area had the highest pollutant loads, followed, respectively, by the eastern, northern, and western parts; (3) the decoupling relationship between ANPSP emissions and agricultural output values showed fluctuating changes, dominated by weak and strong decoupling status, with gradual improvement. (4) Henan Province was divided into three primary non-point source pollution control zones using cluster analysis, namely high, moderate, and low-risk zones. The high, moderate, and low risk areas had average equivalent pollution indices of 61.89, 40.44, and 15.37, respectively. In this study, we proposed targeted prevention and control measures for ANPSP in Henan Province. These findings provide a reference for the governance and planning of ANPSP in Henan Province, as well as a novel perspective for investigating the relationship between rural development and the environment.

## Introduction

Agriculture plays an important role in human sustenance and development. However, the continuous expansion of agriculture production, industial and domestic purposes has resulted in increasingly severe water pollution. As great agricultural country, the use of fertilizers and pesticides in China continuously increase, which enter water bodies through mechanisms such as rainfall, irrigation, or wind during agricultural practices form non-point source pollution (NPSP) [1]. According to the “First National Pollution Source Survey Report of China” (2009), nitrogen and phosphorus from agriculture account for 57.2% and 67.4% of the water pollution, respectively, which has leaded to water eutrophication and affected the ecological environment of water bodies.

In recent years, pollution regulation enhancement and public environmental awareness in China have significantly improved urban living environments through the implementation of effective point source pollution control [2]. However, the governance of NPSP has become a critical challenge [3]. The 2022 National Ecological Environment Status Bulletin highlighted the issue of NPSP, particularly in agricultural areas. Agricultural non-point source pollution (ANPSP) represents the migration of dissolved or solid pollutants (e.g., nitrogen, phosphorus, pesticides) from agricultural areas into water bodies through surface runoff, agricultural drainage, and subsurface infiltration, leading to water quality degradation. Human activities, such as agricultural cultivation and livestock breeding, have resulted in pollutant discharge, contributing significantly to ANPSP and associated greenhouse gas emissions, further aggravating environmental and climatic concerns [4]. In response, collaborative governance mechanisms are increasingly seen as essential to managing complex pollution dynamics such as ANPSP, especially when aligned with adaptive environmental policies [5]. ANPSP can lead to water quality deterioration and eutrophication, seriously affecting the functions of water bodies and the sustainable development of human beings [6]. Numerous researchers have recognized ANPSP as a key issue affecting global water quality [7–9].

China is the world’s largest developing country and a major agricultural country, with large agricultural areas. China has experienced huge population growth and improved living standards since the implementation of reform and opening-up policies, leading to increased demand for grain and livestock meat. To meet these needs, chemical fertilizer applications exhibited a 5-fold increase. Moreover, the 2021 China Rural Statistical Yearbook highlighted a 1.57-fold increase in the number of animals to meet the demand of the livestock and poultry industries (https://www.stats.gov.cn/). The current utilization rate of chemical fertilizers is estimated at 40.2%. On the other hand, the comprehensive utilization rate of livestock and poultry waste is about 75%, while the rural life sewage treatment rate is below 30% (Ministry of Agriculture and Rural Affairs of the People’s Republic of China, 2021). China’s agricultural development depends mainly on the application of large production-related input amounts, resulting in increased crop yields with low utilization efficiency. This issue has resulted in the discharge of chemical fertilizers and livestock/poultry waste into water bodies via surface runoff, leading to serious ANPSP of water environments [10–11]. In addition, Total chemical oxygen demand (COD), total nitrogen (TN), and total phosphorus (TP) are the major pollutant parameter that affects aquatic respiration and the environment if not properly treated before discharge. Consequently, the physicochemical parameter must be reduced to comply with the prevailing effluent discharge standard to ensure environmental sustainability [12]. The Bulletin of the Second National Pollution Source Census outlined substantial contributions of agricultural sources to environmental pollution in China, accounting for 50, 47, and 67% of COD, TN, and TP discharges, respectively [13–14]. The diffuse nature of ANPSP makes related mitigation efforts challenging, posing severe environmental risks. Therefore, effective ANPSP management is crucial for ensuring sustainable development.

Since 2000, China has formulated several sustainable development strategies to improve the quality of its aquatic environments. Since The State Council approved the “Water Pollution Prevention and Control Plan for the Huai River Basin and the Ninth Five-Year Plan” in the 1990s, five phases of water pollution prevention and control plans for key river basins have been compiled and implemented. In addition, the state has continuously issued documents such as the “Action Plan for Water Pollution Prevention and Control”, the “Implementation Opinions on Winning the Battle Against Agricultural Non-point Source Pollution”, and the “Trial Implementation Plan for the Governance and Supervision and Guidance of Agricultural Non-point Source Pollution” in response to water pollution prevention and control. The implementation of the “13th Five-Year Plan for Ecological and Environmental Protection” and the “14th Five-Year Plan for Ecological and Environmental Protection” as a continuation of water pollution control in river basins has given unprecedented high attention to the protection of water ecological environment in river basins. This fully demonstrates the significance and urgency of water environmental pollution in river basins in ecological and environmental protection, and also reflects China’s firm determination to win the battle for clear waters. However, there is still a lack of comprehensive and robust quantitative assessments of sustainable development strategies. Therefore, the assessments of ANPSP-associated risks in China, including their changing trends and spatial distribution characteristics, are required. Specifically, it is crucial to quantitatively explore ANPSP to ensure effective pollution control management [15].

Non-mechanistic and mechanistic modeling methods are the two main approaches used to estimate NPSP [15]. Indeed, numerous models have been used to assess NPSP at various spatial scales, including mechanistic models (e.g., SWAT [16], AGNPS [17], ANSWERS [18]) and empirical models (e.g., inventory analysis [19], export coefficient method [20], discharge factor method [21]). In addition, the assessment of ANPSP loads in farmlands was comprehensively conducted at field crop scales, such as rice, wheat, and maize [22]. However, the application of these pollution parameters remains challenging at watershed or regional scales. The soil and water assessment tool (SWAT) model was extensively employed at the micro-watershed scale to assess watershed ANPSP loads [23]. However, the application of this model in large-scale regional studies is challenging as it requires extensive measured data for its calibration and validation. On the other hand, the export coefficient method has demonstrated a good ability to estimate pollution loads at the county scale and under different land-use types [24]. Although statistical modeling is often less accurate than simulation models, it has been extensively used in NPSP estimation studies, particularly in developing countries where data are often unavailable. Ge et al. [19] employed inventory analysis to quantify ANPSP loads in Guangdong Province over two decades, while Ding et al. [25] investigated emission and migration patterns of cropland pollution in the Dongting Lake Plain. Fang et al. [26] utilized the export coefficient model to identify critical TN source areas and their driving factors.

In recent years, ANPSP has become a complex environmental issue, derived from various sources. Multidimensional research on ANPSP has been conducted, focusing on its current status and driving mechanisms [27], influencing factors, mitigation strategies [28], and relationships with economic growth [29]. These studies have explored ANPSP at different scales, including national [30], watershed [31], and provincial scales [32]. The decoupling concept, originally proposed by the OECD (1993) to describe the dissociation between environmental pressures and economic growth, was modified by Tapio (2005) into an operational evaluation framework [33]. Decoupling has been extensively applied in carbon emissions, water resources, and land use studies [34–35]. In contrast, it remains underexplored in studies on long-term ANPSP-agricultural output relationships in Henan Province.

Henan Province is an important region for modern agriculture and grain production. However, it has been facing severe ANPSP-related challenges due to the impacts of multiple factors, such as national policies, natural ecology, economic level, and population demand. Each district in Henan has its own unique natural environment and socio-economic development characteristics, and the characteristics of ANPSP vary differently. Due to the high difficulty of the investigation, most of the existing studies on ANPSP in Henan have focused on small watersheds. Research on the spatio-temporal characteristics of ANPSP throughout the entire Henan Province at the municipal scale is relatively rare. In this context, the present study aims to estimate ANPSP loads and their spatiotemporal variations in Henan Province, China, over the 2001–2023 period, taking into account different agricultural pollution sources, using a multi-method approach. The specific objectives of this study are to: 1) assess pollutant emissions and identify their sources across prefecture-level cities using long-term statistical data (2001–2023), inventory analysis, and equal standard pollution load method; 2) explore the spatiotemporal patterns of ANPSP; 3) evaluate the dynamic relationship between ANPSP and agricultural output growth using the Tapio decoupling model; 4) explore pollution regional control using cluster analysis. The findings of this study provide information on critical pollution hotspots, as well as a reference for the prevention and control of agricultural non-point source pollution and the sustainable development of the agricultural economy in Henan Province.

## Materials and methods

### Study area

**Fig 1:**
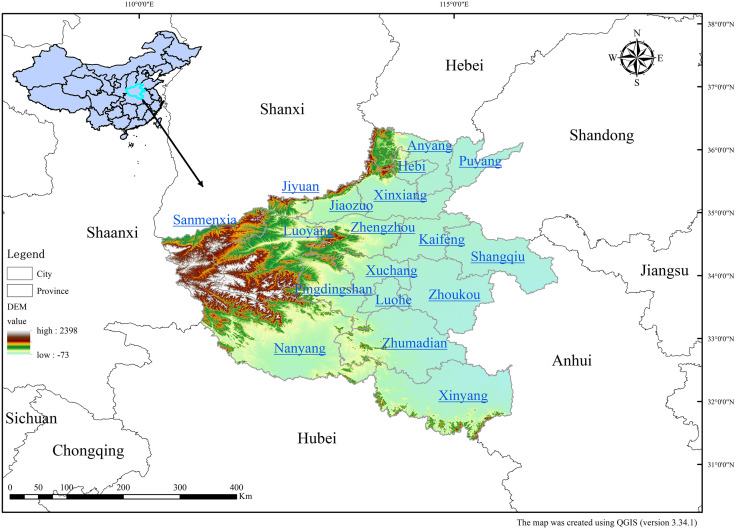
Location map of study area of Henan Province, China.

Henan Province (31°23′-36°22′N; 110°21′-116°39′E) is a pivotal agricultural hub in the Yellow River Basin, covering 167,000 km² in central China’s transitional ecotone between the subtropical and warm-temperate monsoonal climate zones (Fig 1). The province is characterized by three topographic structures, ranging from western highlands (>800 m) to central hills (200–500 m) and eastern alluvial plains (<50 m). This feature makes the province vulnerable to nutrient transport. The semicircular mountain barrier (Taihang-Funiu-Tongbai-Dabie ranges) along the northern, western, and southern boundaries controls the spatial distribution of watershed areas. This results in 92% of runoff water flowing into four major basins, namely Haihe (18% area), Yellow River (21%), Huaihe (53%), and Yangtze (8%).

The Huang-Huai-Hai Alluvial Plain is the dominant agricultural area in the province, covering 82,300 km² (49.3%). It is characterized by the presence of intensive cropping systems, with multiple cropping indices >1.8 and fertilizer inputs at 350 kg N/ha/yr on average. On the other hand, terrace farming is practiced in the western highlands (44,500 km², 26.6%), where comparatively lower chemical inputs are applied (N application <180 kg/ha/yr). This area is also characterized by higher erosion risks, resulting in soil loss of 2,500–3,800 t/km²/yr. The study area includes a transitional Nanyang Basin, with a surface area of 23,200 km² (13.9%). It exhibits mixed paddy-upland systems with intermediate vulnerability.

Hydrologically, the Huaihe Basin has a dense hydrological network in the central-southern parts, receiving 68% of provincial agricultural effluents. At the same time, the Yellow River (711 km) drains 36,200 km² of high-input farmland. The mean annual precipitation in the basin ranges from 500 to 1,200 mm/yr, of which over 70% of the precipitation amount occurs in June-September. These climatic conditions are conducive to spatiotemporal disparities in pollution dilution rates. The northern alluvial aquifers (<15 m) present higher nitrate (NO_3_^-^) contamination risks than southern deep bedrock systems. Indeed, the groundwater NO_3_^-^ concentrations in the alluvial aquifers range from 15 to 28 mg/L.

### Research materials

In this study, an agricultural input and output database was established to estimate the ANPSP loads in Henan Province. The database includes the total annual grain yield and meat output in Henan province, the application amounts of nitrogen, phosphate, and compound fertilizers; the numbers of slaughtered pigs and poultry; the total cattle and sheep, freshwater and marine production, the yields of rice, wheat, corn, vegetables, beans, tubers, and oil-bearing crops, as well as the total rural population from 2001 to 2023. In addition, the annual gross values of agriculture, forestry, animal husbandry, and fishery (AFAF) were included in the database of eighteen cities within Henan Province.

Primary agricultural data were sourced from the Henan Statistical Yearbook (2002−2024). Such as date of the application amounts of nitrogen and phosphorus fertilizer, livestock population, aquaculture yield, and rural population. The coefficient for nitrogen fertilizer and phosphorus fertilizer loss is from the “Manual of Fertilizer Loss Coefficients from Agricultural Pollution Sources”. livestock manure utilization rates were obtained from the China Statistical Yearbook of Animal Husbandry and Veterinary. The contents of COD, TN and TP in the crops refer to “The Chinese Organic Fertilizer Nutrient Catalogue”. Export coefficients for Rural household waste were derived from the Pollution Generation and Emission Coefficient Manual (Agricultural Sources) issued under “China’s Second National Pollution Source Census”; The coefficient of pollution generation in aquaculture is based on the “Manual of Pollutant Generation Coefficients for Aquaculture Industry”. Surface water resource volumes were extracted from the Henan Water Resources Bulletin. Data were collected at annual resolution, with missing values in specific year’s supplemented using linear interpolation. This paper refers to the Environmental Quality Standards for Surface Water (GB 3838−2002), and takes COD, TN, TP as the pollutant concentration discharge standard of class Ⅲ water quality index 20 mg/L, 1.0 mg/L,0.2 mg/L. Geographical datasets, including administrative boundaries, were acquired from the Resource and Environment Science and Data Center, Chinese Academy of Sciences (https://www.resdc.cn/).

### Research methods

#### Inventory analysis method.

Inventory analysis constitutes a systematic quantification methodology that examines resource/energy consumption and environmental emissions, including gaseous discharges, wastewater effluents, solid wastes, and ancillary environmental releases-across the entire life cycle of a product. The methodology encompasses four key components: pollution source identification and adjustment, assessment unit delineation, determination of production and emission coefficients, and pollutant accounting. This method requires the identification of the elementary units (EUs) of agricultural pollution sources. Generally, the agricultural pollution sources in China include three categories. Specifically, the first category includes non-point source (NPS) pollution in farmland that is derived from the applications of mineral fertilizers, plastic film, and pesticides, as well as from straw burning and stacking. The second category consists of NPS pollution from breeding, such as livestock, poultry, and aquaculture breeding. The third category is related to NPS pollution from rural living, such as residential sewage and garbage. In this study, eighteen cities within Henan Province are designated as spatial accounting units to calculate emission magnitudes of five prioritized agricultural pollutants. The primary objective is to delineate spatiotemporal distribution patterns of agricultural NPSP from 2001 to 2023. This study establishes an accounting framework covering five pollution sources: Mineral fertilizers, Livestock and poultry breeding, aquaculture, Farmland straw, and rural household waste. Key indicators including The application amounts of nitrogen and phosphorus fertilizer, livestock population, aquaculture yield, and rural population size were selected to quantify agricultural pollution emissions. Due to data unavailability at the city level, plastic film usage was excluded from the current calculation system, though future studies should consider incorporating these pollution sources.

According to Chen et al. (2006) [36], an EU list of a four-level structure composed of activity, class, unit, and indicator was selected and reported in Table 1. The relationships between activities and total pollution loads (E) were established in this study using a top-down approach, according to the following equation:

**Table 1:** Units of non-point source agricultural pollution.

Activities	Classes	Units	Indicators
Mineral fertilizers	Nitrogen fertilizer (NF)	NF applied to grain crops	Total consumption (10^4^t)
		NF applied to vegetables	
		NF applied to other crops	
	Phosphate fertilizer (PF)	PF applied to grain crops	Total consumption (10^4^t)
			PF applied to vegetables
			PF applied to other crops
Livestock and poultry breeding	Livestock	Cattle	Total annual number (10^4^head)
		Sheep	
		Swine	Number of animals slaughtered (10^4^head)
		Poultry	Poultry
Aquaculture	Freshwater aquaculture	Fish	Fish output (10^4^t)
		Crustaceans	Crustacean output (10^4^t)
		Shellfish	Shellfish output (10^4^t)
		Others	Others output (10^4^t)
Farmland straw	Grain crops	Rice	Yield (10^4^t)
		Wheat	
		Corn	
		Beans	
		Tubers	
	Economic crops	Oil-bearing crops	
	Horticulture crops	Vegetables/ fruits	
Rural household waste	Domestic sewage	Person	Rural population (person)
	Living manure	Person	


$$\text{E}=\sum_{i}EU_{i}\rho_{i}\left(\text{1}-\eta_{i}\right)C_{i}\left({EU}_{i},S\right)=\sum_{i}PE_{i}\rho_{i}\left(\text{1}-\eta_{i}\right)C_{i}\left({EU}_{i},S\right)$$ (1)

where *E* denotes the emission of ANPSP entering the water systems; *EUi* is the statistical index of unit *i*; *ρi* denotes the pollution-generation intensity coefficient of pollutants in unit *i*; *ηi* denotes a coefficient representing the utilization efficiency of relevant resources; *PEi* denotes the maximum amounts of the generated agricultural and rural pollution levels without considering overall resource utilization and management factors. Only pollutants purified by the ecological environment and those entering water bodies and surrounding areas were considered; *Ci* denotes the emission coefficient of pollutants in unit *i*, determined by the *EU* unit i and spatial characteristic *S*, representing the comprehensive impacts of regional environment, rainfall, hydrology, and various management measures on agricultural and rural pollution.

The export coefficient estimates the potential pollution loads of NPSP within a study area by multiplying the average pollutant emission intensity with total activity data. National census data inherently possesses city-level compatibility, as both its institutional design and data collection processes are structured with cities as the fundamental operational units. This study determines export coefficients through the “Second National Pollution Source Census”. As the national census coefficients are updated per decade, the 2017 coefficients from China’s Second National Pollution Source Census were adopted to represent the entire study period (2001–2023).

#### Equal standard pollution load method.

Equal Standard Pollution Load (ESPL) method operates on the principle of comparing the total emission quantity of a given pollutant from various sources against its regulatory discharge standard. Specifically, ESPL quantifies the volume of medium required to dilute emitted pollutants to their respective standard limits, thereby enabling: Assessment of relative hazard levels across pollution sources and contaminants. Comparable evaluation of environmental impacts from disparate pollutants/sources. Concurrently, ESPL quantifies aquatic ecosystem impacts per unit watershed area.

Due to the differences between the pollutant levels and related environmental standards, the pollution-contribution degrees cannot be compared on the same scale. Hence, ESPL was calculated in this study using the following formula:


$$P_{i}=\frac{Q_{i}}{C_{i}}$$ (2)

where *Pi* denotes the equal-standard emissions of ANPSP; *Qi* denotes the annual emissions of pollutants; *Ci* denotes the environmental quality standard of this pollutant. According to the Surface Water Environmental Quality Standard (GB 3838−2002), the Class Ⅲ standard limits of COD, TN, and TP were set at 20, 1.0, and 0.2 mg/L respectively.

Although the equal-standard emissions can be compared on the same scale, it considers the total amount. They can be affected by land area, agricultural population, and total agricultural output value. The pollution discharge intensity *EI* was calculated in this study using the following formula:


$$\text{EI}=\frac{E}{AL}$$ (3)

where *AL* denotes the output value; *EI* denotes the pollution discharge per 10,000 yuan of the output value of a specific agricultural non-point source pollutant. Pollution discharge/10,000 yuan of output value is an emission indicator widely used, reflecting the relationship between the environment and the economy; *AL* denotes the agricultural land area of the study region; *EI* represents the emission intensity of ANPSP, reflecting the pollutant concentration level. In addition, this parameter can be used to reflect agricultural intensification levels in a region and associated impacts on water bodies. *AL* represents the surface water resource quantity in the study region; *EI* denotes the emission concentration of ANPSP, reflecting its impact on the water environment.

#### The Tapio decoupling evaluation model.

The reasons for introducing Tapio decoupling analysis are (1) to carry on the above calculation results to better identify and compare the relationship between environmental consumption or pollution emssions and economic growth in Henan province, while the traditional EKC test fails to identify the relationship between the two at a specific stage; (2) most of the decoupling researches focus on carbon emissions, water resources, and land resources [37], while the decoupling research on ANPSP and agriculture output values is relatively rare; (3) higher sensitivity than the decoupling indicator method, developed by the Organization for Economic Co-operation and Development (OECD). This model can, indeed, accurately reflect the decoupling relationship between ANPSP and output values. The decoupling states can be divided into eight categories according to the sign direction of variables and the values of elasticity coefficients (Table 2). Among them, high decoupling rates indicate the most optimal agricultural development status, indicating the lack of high pollutant discharge rates. The decoupling elasticity coefficient was calculated in this study using the following equation:

**Table 2:** Categories of the decoupling rates.

∆I	∆G	W	Categories
>0	<0	<0	Strong negative decoupling
<0	<0	0 ≤ W < 0.8	Weak negative decoupling
>0	>0	1.2 ≤ W	Expansive negative decoupling
<0	<0	0.8 ≤ W < 1.2	Recessionary coupling
>0	>0	0.8 ≤ W < 1.2	Growth coupling
<0	<0	1.2 ≤ W	Recessive decoupling
>0	>0	0 ≤ W < 0.8	Weak decoupling
<0	>0	<0	Strong decoupling


$$\text{W}=\frac{\Delta I}{\Delta G}=\frac{I_{t}/I_{t-\text{1}}-\text{1}}{G_{t}/G_{t-\text{1}}-\text{1}}$$ (4)

where *W* denotes the elasticity coefficient between ANPSP and agricultural output value; Δ*I* and Δ*G* denote the changing rates of pollutant emissions and agricultural output value, respectively; *I*_*t*_ and *I*_*t*−1_ represent the pollutant emissions in year *t* and year *t* − 1 in 10,000 tons, respectively; *G*_*t*_ and *G*_*t*−1_ represent the agricultural output value in year *t* and year *t* − 1 in 100 million yuan, respectively.

#### K-means clustering analysis.

Data clustering algorithms are generally categorized into two main types: hierarchical clustering and partitional clustering [38]. K-means clustering is a popular and efficient partition-based clustering algorithm. It generates clusters using a heuristic approach while optimizing a standard function defined globally in the dataset or locally within subsets of data objects [39]. K-means clustering operates by partitioning a dataset into clusters while minimizing the squared error between each sampling point and its assigned cluster centroid. The algorithm measures variance based on a concept of distance that reflects the similarity between two samples, rather than traditional spatial distance. The choice of distance metric significantly influences the assessment of similarity [40], with the standard K-means algorithm commonly employing the Euclidean metric as its default distance measure.

In our study, the Euclidean distance was set as the measurement standard using SPSS 26.0 software based on the calculation results of the equivalent standard pollution index. We adopted the partitional cluster analysis (K-means clustering) based on the sum of squared deviations. By using the equivalent standard pollution indices of various pollutants as statistical variables, cluster analysis was carried out to generate a dendrogram, through which study units with similar pollution levels were grouped into the same category. According to the clustering results, different levels of non-point source pollution control zones for the study area were derived.

## Results

### Characteristics of ANPSP

**Fig 2:**
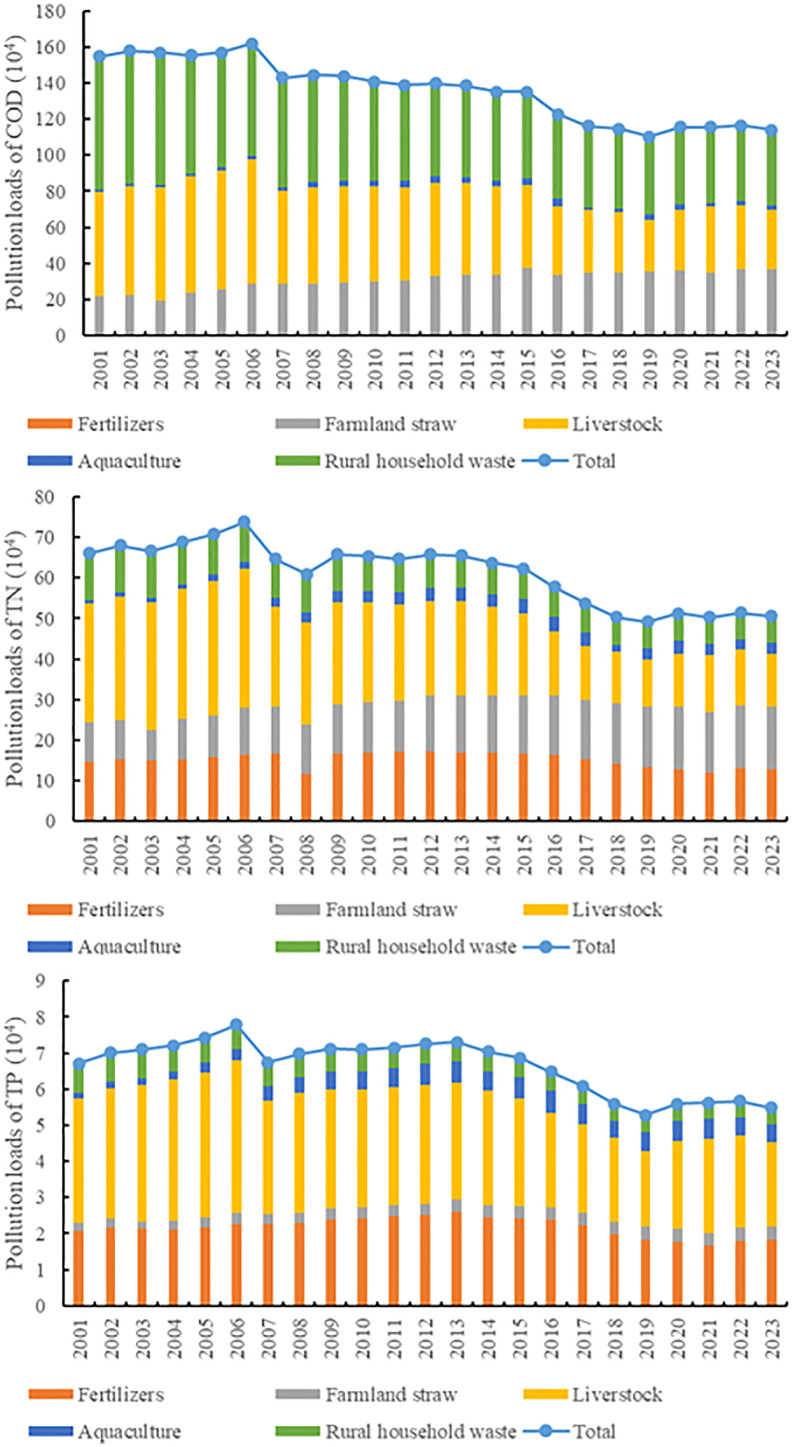
Pollution loads of different agricultural non-point sources pollutants between 2001 and 2023 in Henan Province, China.

The results showed decreasing trends in the COD, TN, and TP concentrations (Fig 2). The 2001–2023 period can be divided into four typical stages according to the dynamic variations in the ANPSP loads:

(1) 2001–2006: Agricultural Expansion with Accelerated pollutant discharge

The total ANPSP loads showed an increasing trend over this period. Specifically, the COD emissions increased from 154.69 × 10^4^ t to 161.85 × 10^4^ t, corresponding to a 4.6% increase. From 2001 to 2004, the proportion of rural household waste was above 40%, making it the main source of COD emissions in the study area. From 2005 to 2006, the proportion of livestock exceeded that of rural household waste, becoming the main source of COD emissions. On the other hand, the proportion of rural household waste decreased from 47.8 to 38.62% over the 2001–2006 period, while the proportion of livestock increased from 37.4 to 42.7%. This finding might be related to the rapid expansion of large-scale poultry farming in 2006. Specifically, the livestock and poultry inventory in 2006 increased by 28% compared with that in 2001, with low manure treatment and resource utilization rates (below 20%), resulting in prominent direct pollutant discharge. The TN emissions increased from 66.14 × 10^4^ t to 73.82 × 10^4^ t, representing a growth rate of 11.6%. Livestock was the main source of TN emissions, accounting for about 45%, followed by that of fertilizers (22%). The utilization rate of farmland straw increased from 14.5 to 15.7%. This finding might be related to the promotion of high-fertilizer-consuming crops, such as wheat and corn, by the grain direct subsidy policy in 2004, resulting in an annual increase in the fertilizer applications by an average proportion of 3.2%. Furthermore, the increase in the farmland straw proportion might be attributed to an increased loss of N due to open-air burning of straw. The TP emissions increased from 6.71 × 10^4^ t to 7.79 × 10^4^ t, representing a growth rate of 16.1%. Livestock was the main source of TP emissions, with an increased contribution rate from 51.3 to 54.4%. This finding might be caused by the low coverage rate of manure treatment facilities through the livestock and poultry intensification processes.

Official data from the Henan Provincial Environmental Status Bulletin indicate that water quality trends during this period were linked to ANPSP. Key pollutants-ammonia nitrogen (NH₃-N), COD, and BOD-closely aligned with agricultural emissions from crop and livestock activities. Although overall water quality improved from “heavily polluted” to “lightly polluted” (with the composite pollution index declining from 0.62 to 0.52), indicating effective point source control, the proportion of Grade IV and V water segments increased from 20% to 21.3%. This rise in lightly to moderately polluted water bodies corresponds to the diffuse and persistent nature of agricultural NPS pollution, highlighting its growing relative contribution. Thus, while point source reductions led to general improvement, agricultural NPS pollution has become a key constraint to further water quality gains, underscoring the need for enhanced management strategies.

(2) 2007-2013: Policy-Driven Structural Transitions

The total amount of pollutant emissions in the 2007–2013 period showed a downward trend. The COD emissions decreased from 161.85 × 10^4^ t to 138.49 × 10^4^ t, corresponding to a reduction rate of 14.43%. Although rural household waste exhibited a continuous decrease during this period, it was the main source of COD emissions. The proportions of rural household waste and livestock were similar in the 2012–2023 period at about 35%. This finding might be influenced by the promotion of household biogas tank construction as a part of the 2007 Henan Province Rural Environmental Protection Plan, as well as by the closure of small-scale livestock farms due to the implementation of the Livestock Breeding Pollution Prevention and Control Regulations in 2011. The TN emissions decreased from 73.82 × 10^4^ to 60.94 × 10^4^ t, and then increased to 65.75 × 10^4^ t. Livestock was the main source of TN emissions. The proportion of livestock and rural household waste exhibited continuous decreases, while the proportion of fertilizers, farmland straw, and aquaculture showed increasing trends. The proportions of fertilizers, farmland straw, and aquaculture increased from 22.2 to 25.9%, 15.7 to 21.7%, and 3.4 to 5.1%, respectively. These findings reflect the low straw return rates and the intensification of fertilizer losses. The occurrence of a rainstorm event in South Henan Province in 2010 might also have led to an increase in TN emissions from agricultural runoff, increasing ecological vulnerability. The TP emissions decreased from 7.79 × 10^4^ to 6.74 × 10^4^ t and then showed a continuous upward trend. Livestock was the main source of TP emissions. However, its proportion continued to decline from 54.4 to 44.4%. In contrast, the proportion of aquaculture increased from 4.0 to 7.9%. This might be related to the expansion of intensive aquaculture areas.

During the period from 2007 to 2013, while agricultural non-point source pollution emissions in the region showed a declining trend, water quality did not exhibit synchronous improvement. Instead, it displayed clear hysteresis and spatial heterogeneity. In 2007, the overall surface water quality was classified as lightly polluted, with a comprehensive pollution index of 0.52; by 2013, it had deteriorated to moderately polluted. Although the proportion of Grade I-III water sections increased from 53.4% to 45.8% of the total monitored length, the proportion of inferior Grade V sections remained high, decreasing only from 32.3% to 24.1%. Notably, the Huai River Basin experienced a decline from light to moderate pollution, while the Hai River Basin consistently maintained severe pollution levels. Throughout this period, key pollution indicators-ammonia nitrogen (NH₃-N), COD, and five-day biochemical oxygen demand (BOD₅)-remained characteristic of agricultural non-point source pollution. The persistence of these pollutants, coupled with the lagged and uneven water quality response, suggests that the reduction in agricultural emissions alone was insufficient to fully restore water quality. This may be attributed to legacy pollution accumulation, continuous input from other sources, and the inherent complexity of non-point source pollution control. These findings underscore the necessity of adopting long-term, integrated river basin management strategies that account for temporal lags and spatial variability in environmental responses.

(3) 2014-2019: Technology-Enhanced Emission Control

The total pollution loads in the 2014–2019 period showed a continuous downward trend. The COD emission decreased from 138.49 × 10^4^ to 110.23 × 10^4^ t, corresponding to a reduction rate of 20.4%. Rural household waste was the main source of COD emissions, with a proportion rate of 38.9%. This result indicates the delayed centralized sewage treatment in rural areas following the increase in the urbanization rate. The contribution rate of livestock decreased from 36.2 to 26.2%, which might be attributed to the implementation of the Zero Growth Action on Fertilizers in 2015 and the policy of improving the utilization rate of livestock and poultry manure and its resources. The TN emission decreased from 65.46 × 10^4^ to 46.26 × 10^4^ t, corresponding to a reduction rate of 29.33%. Farmland straw was the main source of TN emissions. The proportions of fertilizers and livestock decreased to 26.9 and 23.4%, respectively. This was partly related to the “Grain-to-Fodder” policy, promoting integrated farming and breeding. However, the occurrence of continuous drought events from 2016 to 2018 reduced surface runoff, indirectly reducing nitrogen loss intensities. The TP emission decreased from 7.31 × 10^4^ to 5.29 × 10^4^ t, representing a reduction rate of 27.63%. Livestock was the main source of TP emissions in the 2014–2019 period, even though its proportion decreased from 44.9 to 39.5%. On the other hand, the proportion of farmland straw sources increased from 4.9 to 6.8%, suggesting continuous improvement in the comprehensive utilization rate of manure in Henan Province and its emission reduction effects.

Between 2014 and 2019, while ANPSP loads in the region continued a declining trend, water quality exhibited a clear and positive response, indicating gradually effective pollution control. In 2014, the overall surface water quality was moderately polluted, with key pollutants being COD, BOD₅, and TP. By 2019, water quality had improved to lightly polluted, with NH₃-N reappearing as a key pollutant, replacing TP, and the complete elimination of inferior Grade V sections in national monitored sections. The proportion of Grade I-III sections in provincial monitoring increased from 44.6% to 63.1%, demonstrating a clear structural improvement in water quality. The relative stability and subsequent improvement in conventional organic pollutants and nutrients align with the characteristics of ANPSP mitigation, suggesting that sustained reduction in agricultural emissions. The Environmental Index (EI) value has also increased from 53 to 62.6.

(4) 2020-2023: Structural Bottleneck Governance

The total pollution loads in the 2020–2023 period had a relatively constant trend. Specifically, the COD emission loads slightly increased. Rural household waste was the main source of COD loads in this period, followed by livestock and farmland straw. The TN and TP loads were stable. On the other hand, TN and TP loads were derived from farmland straw and livestock, respectively. The stabilization of the total ANPSP revealed governance bottlenecks. The COD loads showed a slight decrease even under a rural sewage treatment rate of 65%. This might be due to the incomplete implementation of a biogas project as a result of insufficient network subsidies. TN showed a similar decreasing trend. However, the occurrence of flood events in 2021 resulted in an increase in the monthly TN load by 40%. The stabilization of TP loads might be related to the reduction in fertilizer application and the lack of organic substitutes as a result of the reduction in smallholder farm exits (45% overloaded manure facilities in 2023).

**Fig 3:**
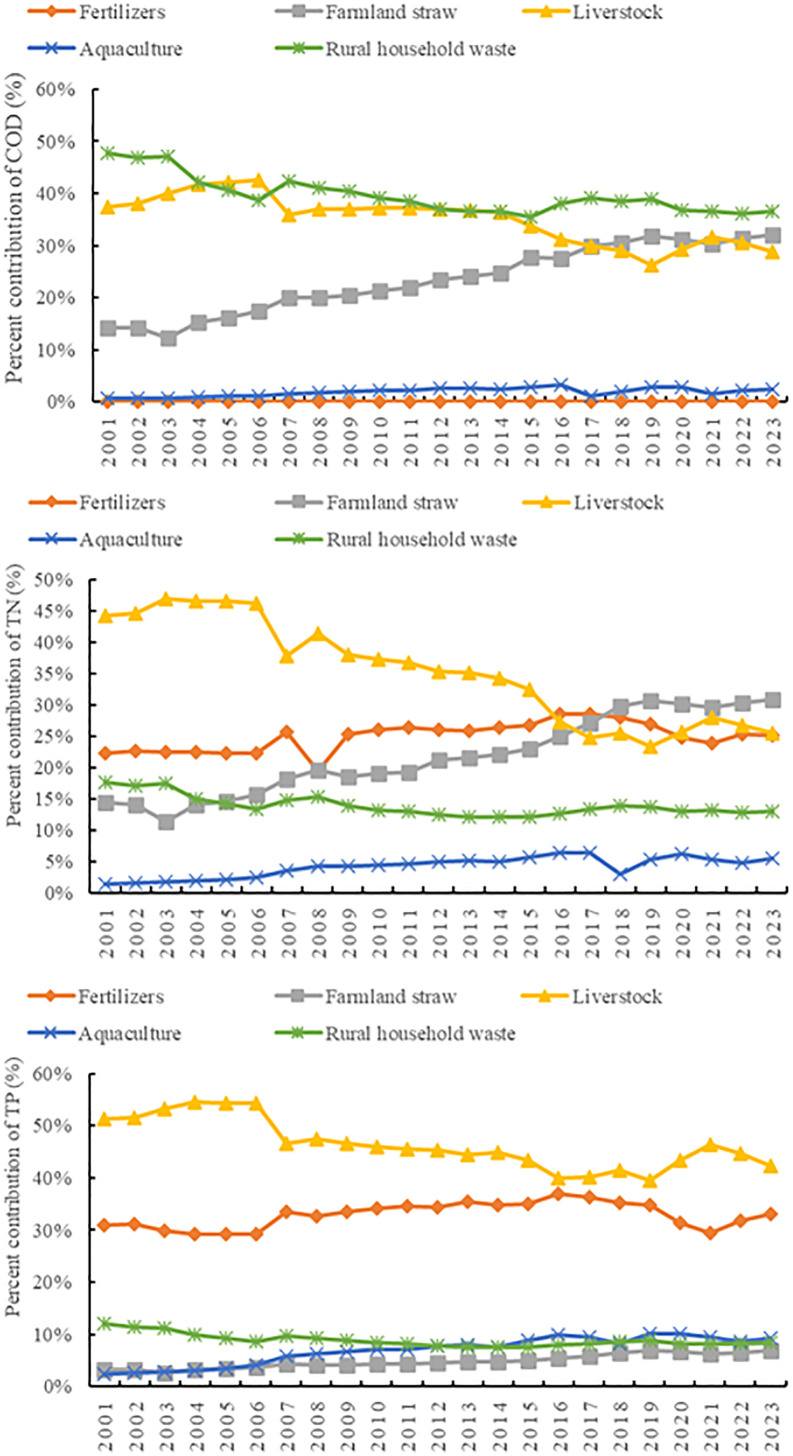
Percent pollution of different agricultural non-point sources pollutants between 2001 and 2023 in Henan Province, China. The y-axis represents the percent contribution to a pollutant from a source to the total amount of that pollutant.

Structural variations were observed in the different ANPSPs in the 2001–2023 period (Fig 3). The main sources of COD were rural household waste, as well as livestock and poultry breeding. Conversely, the contribution of farmland straw-derived COD showed an increase from 14.15 to 32.14% during the 2001–2023 period. Although the main sources of TN were livestock and poultry breeding, their contributions decreased progressively. In contrast, the contribution rate of mineral fertilizers and farmland straw to TN loads increased during the 2016–2020 period, particularly surpassing livestock sources. On the other hand, the contribution of livestock and poultry breeding to TP loads decreased, while that of mineral fertilizers showed an increasing trend. Furthermore, aquaculture emerged as an escalating pollution source, leading to increases in the TN and TP proportions from 1.3 and 2.3% to 5.4 and 9.3%, respectively, over the 2001–2023 period.

Between 2020 and 2022, ANPSP loads in Henan Province remained relatively stable, while water quality continued to demonstrate a positive trajectory, suggesting the sustained benefits of earlier pollution control measures and the growing role of ecological and point source management. In 2020, the overall river water quality was classified as lightly polluted, with key pollutants including COD, permanganate index, and BOD₅. By 2022, the overall water quality had improved to good, with the complete elimination of inferior Grade V sections in national monitoring. The proportion of Grade I-III sections increased from 73.8% to 81.9%, indicating further structural improvement in water quality across the province.

Spatial heterogeneity persisted, with the Hai River Basin remaining lightly polluted while the Yellow, Huai, and Yangtze River Basins all reached good or excellent quality levels. The stability in conventional agricultural pollutants-even amid overall water quality gains-aligns with the plateau in NPS emissions, implying that continued point source treatment and integrated river management have helped drive further improvements. The decline in EI may suggest that broader environmental pressures persist even as water quality recovers, highlighting the need to balance water-specific interventions with basin-wide ecological restoration

Overall, the evolution of ANPSP in Henan Province exhibited three major characteristics: aggregate reduction (18.7% decrease in total loads over the 2001–2023 period), structural rigidity (persistent dominance of conventional pollution sectors), and regional differentiation (eastern regions showing 2.3 times higher pollutant loads than western areas). These dynamics were driven by multifactorial interactions between policy interventions, production mode transitions, and extreme climate events.

### Spatiotemporal patterns of ANPSP

The above-mentioned typical periods were selected to reveal the spatiotemporal patterns of the pollutant loads. The natural break-point was used to divide the load concentrations into five grades. The spatial distribution of total emission and total emission intensity of pollutants exhibited a distinct gradient pattern.

**Fig 4:**
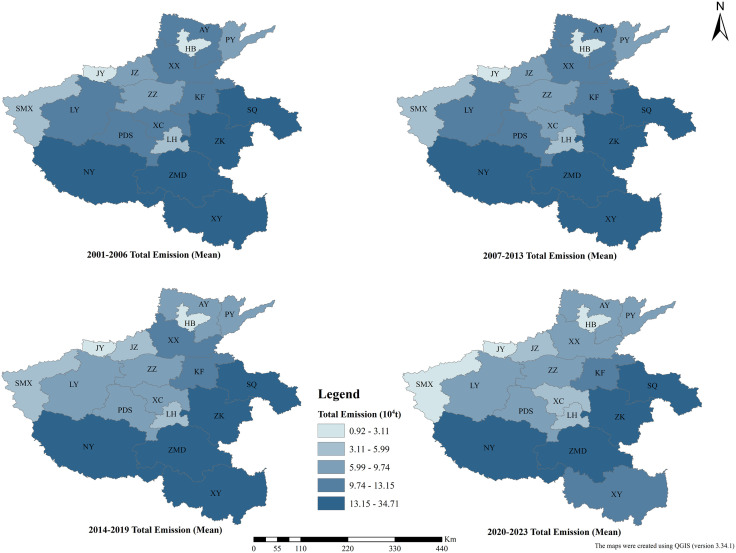
Spatial distribution map of total pollutant emissions in Henan Province.

The highest total emissions were observed in the northern and eastern parts of the study area (Fig 4). On the other hand, the lowest total emissions were found in the northern and western parts. The cities with the highest total emissions are Xinxiang, Anyang, and Puyang, showing average total emission amounts of 11.24 × 10^4^, 10.06 × 10^4^, and 7.35 × 10^4^ t, respectively. In contrast, the cities with the highest total emission amounts in the southern region are Nanyang, Zhumadian, and Xinyang, showing average total emissions of 26.76 × 10^4^, 21.76 × 10^4^, and 15.61 × 10^4^ t, respectively. Luoyang was the city with the highest total emission amount in the western part of the study area, with an average total emission amount of 10.45 × 10^4^t. Whereas Zhoukou, Shangqiu, and Kaifeng were the cities with the highest total emissions in the eastern part, with average total emission amounts of 22.33 × 10^4^, 18.81 × 10^4^, and 11.78 × 10^4^ t, respectively.

**Fig 5:**
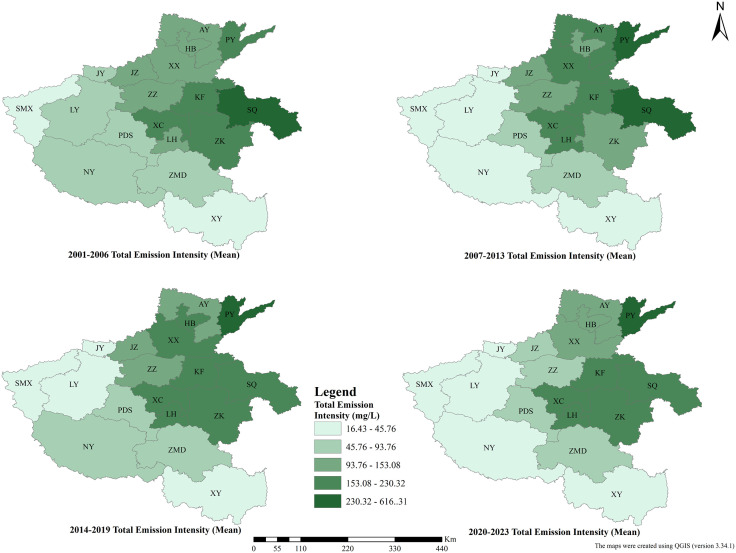
Spatial distribution map of total pollutant emission intensity in Henan Province.

The total pollutant emission intensity showed a distinct spatial gradient pattern, with higher and lower amounts in the northeastern and southwestern parts, respectively (Fig 5). The cities with the highest total emission concentrations in the northern part of the study area were Puyang, Xinxiang, Hebi, and Anyang, with average emission concentrations of 442.73, 239.81, 229.79, and 213.32 mg/L respectively. Whereas the cities with the highest total emission concentrations in the eastern part are Shangqiu, Kaifeng, and Zhoukou, with average emission concentrations of 398.06, 307.96, and 259.23 mg/L, respectively. Xuchang and Luohe in the central part of the study area exhibited comparatively lower average total emissions of 8.34 × 10^4^ and 5.13 × 10^4^ t, respectively. Nevertheless, high average emission concentrations were found in Xuchang and Luohe at 278.76 and 251.06 mg/L, respectively, due to the effects of surface water resources.

The cities with the highest equivalent COD load in the 2001–2006 period were Nanyang, Zhoukou, Zhumadian, Shangqiu, and Xinyang, reaching values of 113.57 × 10^8^, 87.26 × 10^8^, 83.25 × 10^8^, 69.48 × 10^8^, and 57.67 × 10^8^ m^3^, respectively (S1 Appendix in S1 File). The top five cities in terms of equivalent TN loads owere Nanyang, Zhoukou, Zhumadian, Shangqiu, and Xinyang, with values of 1084.26 × 10^8^, 793.42 × 10^8^, 778.91 × 10^8^, 611.85 × 10^8^, and 526.72 × 10^8^ m^3^, respectively (S2 Appendix in S1 File). On the other hand, the top five cities in terms of equivalent TP loads were Nanyang, Zhoukou, Zhumadian, Shangqiu, and Xinyang, with values of 572.32 × 10^8^, 400.8 × 10^8^, 384.91 × 10^8^, 317.4 × 10^8^, and 278.12 × 10^8^ m^3^, respectively (S3 Appendix in S1 File). In fact, the systematic prevention and control policies against large-scale livestock-derived pollution in the 2001–2006 period were not effectively implemented by the national and local governments. Furthermore, environmental supervision was relatively weak without effective pollution prevention and control facilities or policies for waste resource utilization, explaining the high pollutant emissions and the prominent non-point source pollution.

The cities with the highest equivalent COD loads over the 2007–2013 period were Nanyang, Zhumadian, Zhoukou, Shangqiu, and Xinyang, with values of 86.08 × 10^8^, 74.83 × 10^8^, 73.22 × 10^8^, 64.57 × 10^8^, and 53.44 × 10^8^ m^3^, respectively (S1 Appendix in S1 File). On the other hand, Nanyang, Zhumadian, Zhoukou, Shangqiu, and Xinyang had the highest equivalent TN loads of 851.9 × 10^8^, 697.45 × 10^8^, 675.7 × 10^8^, 600.62 × 10^8^, and 590.78 × 10^8^ m^3^, respectively (S2 Appendix in S1 File). Whereas Zhoukou, Nanyang, Zhumadian, Shangqiu, and Xinyang exhibited the highest equivalent TP loads of 545.32 × 10^8^, 449.55 × 10^8^, 379.44 × 10^8^, 340.64 × 10^8^, and 322.93 × 10^8^ m^3^, respectively (S3-S6 Appendix in S1 File). During this period, the pollutant emissions of each city gradually decreased due partially to the implementation of the First Pollution Census and the strengthening of the ANPSP governance, including the delimitation, closure, and relocation of prohibited and restricted breeding areas, as well as the reduction of direct pollutant emissions in Henan Province. The promotion of biogas projects and the promotion of livestock manure utilization effectively reduced the direct emissions of COD and nutrients.

Nanyang, Zhumadian, Zhoukou, Shangqiu, and Xinyang were the main cities with the highest equivalent COD loads in the 2014–2019 period, reaching 75.6 × 10^8^, 66.95 × 10^8^, 63.75 × 10^8^, 59.32 × 10^8^, and 44.99 × 10^8^ m^3^, respectively (S1 Appendix in S1 File). In contrast, Nanyang, Zhumadian, Zhoukou, Shangqiu, and Xinyang had the highest equivalent TN loads of 761.27 × 10^8^, 623.63 × 10^8^, 619.67 × 10^8^, 564.22 × 10^8^, and 509.04 × 10^8^ m^3^, respectively (S2 Appendix in S1 File). The highest TP loads were found in Shangqiu, Nanyang, Zhoukou, Zhumadian, and Xinyang, reaching 397.44 × 10^8^, 395.63 × 10^8^, 348.59 × 10^8^, 324.63 × 10^8^, and 295.59 × 10^8^ m^3^, respectively (S3 Appendix in S1 File). The continuous decreases in the pollutant loads in the 2014–2019 period might be attributed to the stringent and systematic environmental governance policies at the national level. The Regulations on the Prevention and Control of Pollution from Livestock and Poultry Farming Plan was implemented in China to prevent and control the discharge of pollutants from livestock and poultry farming. It imposes mandatory requirements on the environmental assessment of farms, the construction of waste treatment facilities, and resource utilization, greatly promoting the governance process. The Water Pollution Prevention and Control Action Plan was also implemented to prevent pollution from livestock and poultry farming, thereby effectively controlling ANPSP and strengthening the responsibilities and assessment targets of the local governments. In addition, strict agricultural pollution control measures were implemented in the water source areas of Nanyang and the Danjiangkou Reservoir area following the intermediate route of the South-to-North Water Diversion Project. Although the COD pollution index was relatively constant, some cities (e.g., Xinyang and Zhumadian) exhibited high TN and TP pollution indices of 23.09 and 31.42, respectively, due to the occurrence of high agricultural output values and the presence of strong water system connectivity. These pollutants became the main environmental challenges for water quality improvement.

The top five cities in terms of COD loads in the 2020–2023 period were Nanyang, Zhoukou, Zhumadian, Shangqiu, and Xinyang, reaching 72.16 × 10^8^, 67.91 × 10^8^, 63.26 × 10^8^, 57.39 × 10^8^, and 40.93 × 10^8^ m^3^, respectively (S1 Appendix in S1 File). The highest TN loads were found in Nanyang, Zhumadian, Zhoukou, Shangqiu, and Xinyang, reaching 705.77 × 10^8^, 624.48 × 10^8^, 610.19 × 10^8^, 467.41 × 10^8^, and 445.93 × 10^8^ m^3^, respectively (S2 Appendix in S1 File). Whereas the highest TP and other pollutant loads were observed in Nanyang, Zhoukou, Zhumadian, Shangqiu, and Xinyang, with values of 362.85 × 10^8^, 345.04 × 10^8^, 308.7 × 10^8^, 281.62 × 10^8^, and 253.87 × 10^8^ m^3^, respectively (S3 Appendix in S1 File). The continuous decreases in the pollutant loads over the 2020–2023 period might be due to the implementation of the above-mentioned policies and the reinforcement of ANPSP control. Moreover, the advancement of the Rural Revitalization Strategy and the Action Plan for the Battle against Agricultural and Rural Pollution Control in China effectively reduced the high-pollution areas in Henan Province. Cities with high urbanization rates, such as Zhengzhou and Luoyang, experienced a decrease in the pollutant loads due to the reduction of agricultural land. However, the southern region in Henan province had high emission pressure with a long treatment cycle.

### Decoupling the relationship between ANPSP and output value

**Fig 6:**
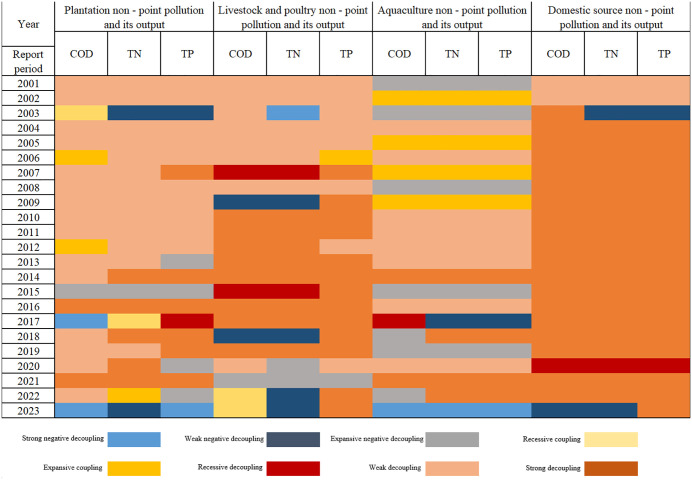
Temporal variation characteristics of the decoupling state of agricultural non-point pollution and output in Henan Province, China.

The elastic decoupling status between ANPSP and agricultural output values showed fluctuating changes, showing weak and strong decoupling status, with obviously spatial variability (Fig 6). The changes in the decoupling status were influenced by multiple factors, such as national policies, agricultural restructuring, economic level, and technological advancements. In this study, sectoral analyses were performed based on the OECD decoupling framework and classification criteria, as follows:

### Non-point pollution from plantations and associated output

The decoupling trajectory displayed marked phase transitions from “weak decoupling dominance” to “strong decoupling advancement”. The plantation-derived COD, TN, and TP loads in the 2001–2006 period predominantly exhibited weak decoupling, indicating synchronized low-rate expansion of pollution with output growth, which is consistent with the increase in the annual grain yields and synthetic fertilizer applications. The agricultural output exhibited an increasing trend from 2007 to 2014, which might be due to the implementation of the National Plan for a 50-billion-kilogram Incremental Grain Production Capacity. The TN and TP loads entered an expansive negative decoupling phase, highlighting intensified pollution pressures from nitrogen leaching in rice paddies and straw burning. Besides organic fertilizer substitution, the Zero-Growth Action for Fertilizers and Pesticides relevantly elevated strong decoupling ratios in 2015. The encouragement of agricultural policy effectively decoupled pollution from economic growth.

### Non-point pollution from livestock/poultry and its output

The paradigm shift from “expansive negative decoupling” to “strong decoupling dominance” highlighted the critical role of policy interventions. The decoupling states of the COD, TN, and TP loads were in weak or expansive negative conditions in the 2001–2009 period due to the implementation of inadequate pollution control infrastructure in the early stage of livestock industrialization. On the other hand, strong decoupling was observed in the 2001–2023 period, accounting for 67%, due to the implementation of regulation policies on pollution prevention in livestock and poultry scale breeding and the Qian Dui Wan Chi project. In addition, 23,000 farms in prohibited zones along the Huaihe River and Yellow River basins were closed after the implementation of the Water Pollution Prevention Action Plan. Subsequently, the decoupling states of the COD, TN, and TP loads in 2017 were strong. Notably, the expansive negative decoupling in 2022 might related to the increase in small-scale agricultural activities and the late supervision of pollution control.

### Non-point pollution from aquaculture and its output

The decoupling status was unstable due to the effect of the breeding mode and environmental carrying capacity. The aquaculture activities in the southern part of Henan Province, where Xinyang and Nanyang are located, experienced expansion over the 2001–2010 period, with an average annual growth rate of 5.8%. However, the widespread direct discharge of wastewater from pond aquaculture has led to environmental concerns, increasing the COD, TN, and TP levels. After 2014, the proportion of areas with weak decoupling increased because of the implementation of sustainable aquaculture demonstration farms and the promotion of wastewater treatment facilities. However, expansive negative decoupling occurred in 2019, indicating the persistence of low utilization rates of facilities and uneven regional supervision. Pollution emissions and output value exhibited a decoupling trend in the 2021–2023 period as a result of the ten-year fishing ban policy on the Yangtze River.

### Domestic non-point source pollution and its output

Maintaining a sustained status of strong decoupling indicates obvious advancements in enhancing the rural living environment. The strong decoupling proportions of COD, TN, and TP from domestic sources all exceeded 80% over the 2001–2023 period, mainly due to the implementation of the Three-Year Action Plan for Improving the Rural Living Environment and Wastewater Revolution projects. The coverage rate of rural sanitary toilets in Henan Province increased from 35 to 90%, while the treatment rate of domestic sewage reached 65%, effectively preventing the discharge of domestic pollutants into rivers. Particularly, a recessionary decoupling was observed in 2023, characterized by a simultaneous reduction in pollution levels and output values. This might be attributed to the decrease in rural population during urbanization by about 15 million rural permanent residents between 2010 and 2020, as well as to the reduction of domestic pollution sources.

### Non-point source pollution control zones

**Fig 7:**
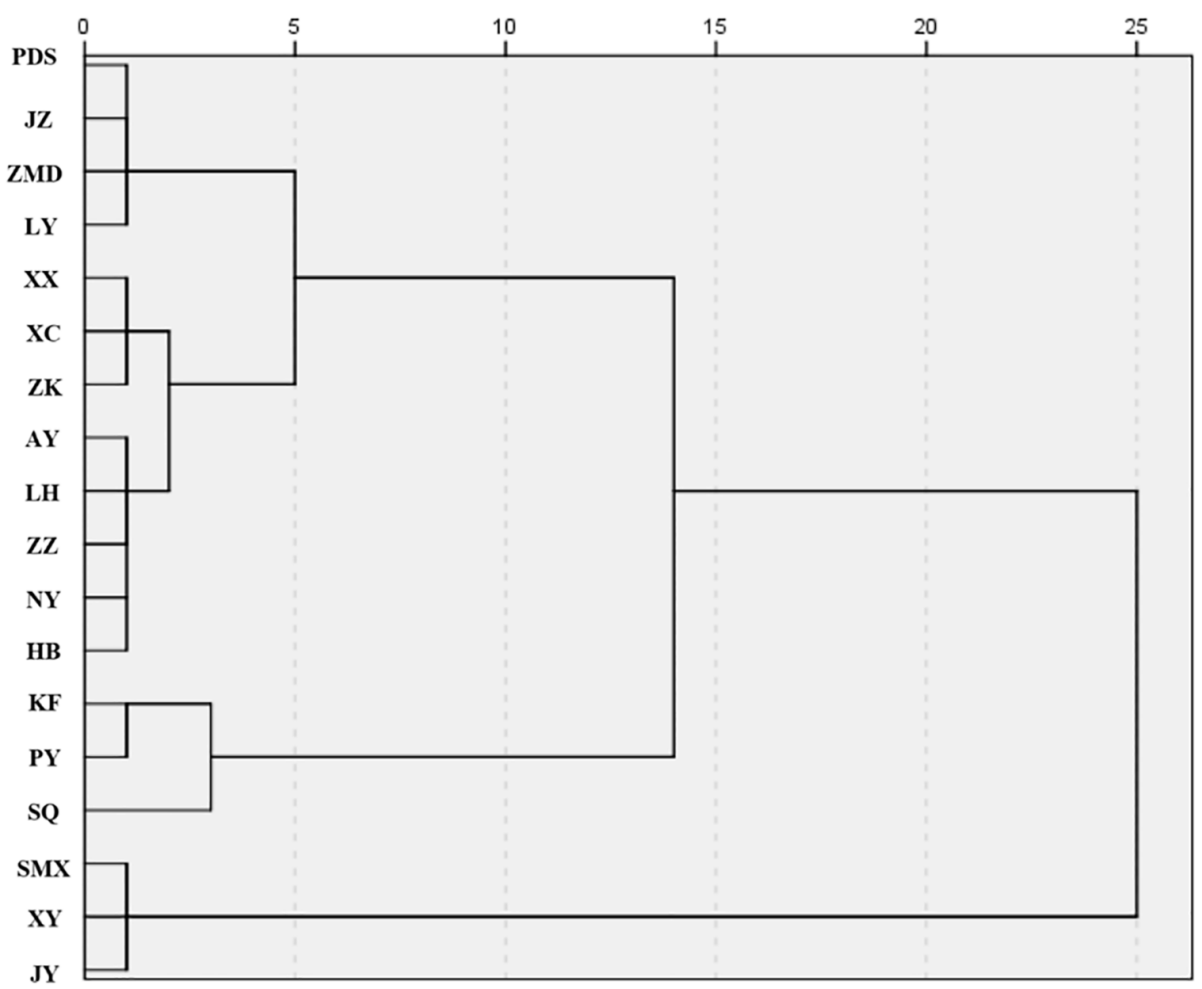
Cluster analysis of ANPSP in 18 prefecture-level cities in Henan Province.

**Fig 8:**
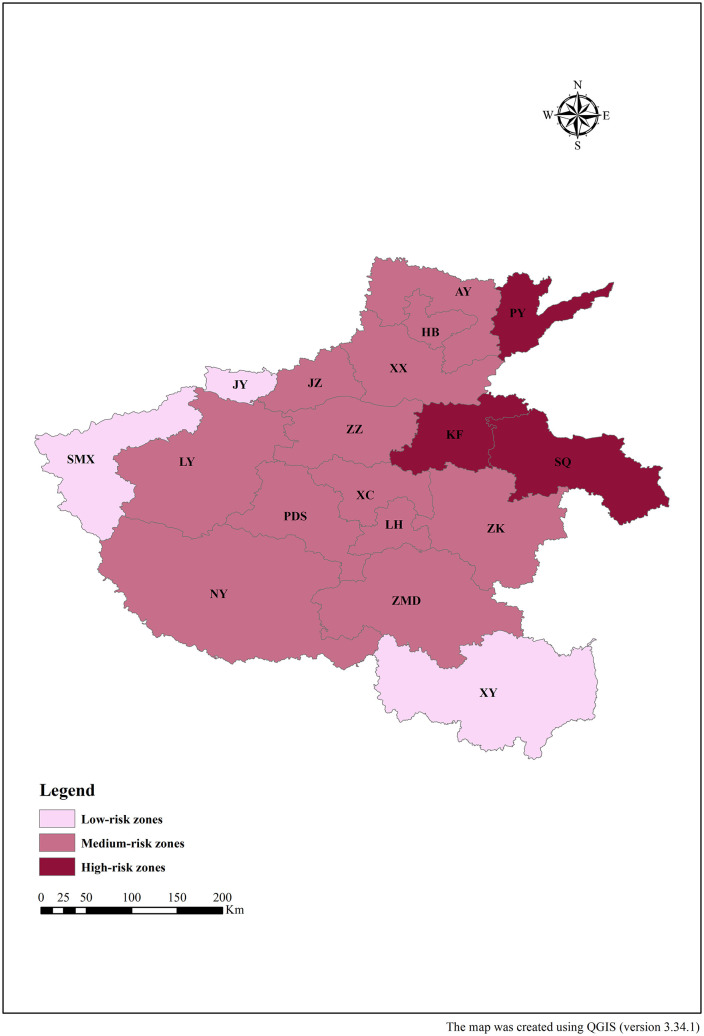
Cluster analysis control zoning map of Henan Province.

In this study, spatial clustering analysis was performed to further assess the equivalent indices of ANPSP in 18 prefecture-level cities in Henan Province. Different pollution levels in the study area were determined from various distances. The obtained results are shown in the Fig 7 and Fig 8. The risk levels of ANPSP in Henan Province were classified at a Euclidean distance threshold of 14 into three categories, namely high, moderate, and low-risk areas (Fig 7). The high-risk zones included Kaifeng, Puyang, and Shangqiu, with an average equivalent pollution index of 61.89 (Fig 8). These areas were affected by various factors, including large-scale livestock and poultry farming, rural population, and intensive agricultural activities (grain, vegetables, and crop cultivation). The moderate risk zones consisted of Pingdingshan, Jiaozuo, Zhumadian, Luoyang, Xinxiang, Xuchang, Zhoukou, Anyang, Luohe, Zhengzhou, Nanyang, and Hebi, with an average equivalent pollution index of 40.44 (Fig 8). The low-risk zones included Sanmenxia, Jiyuan, and Xinyang, with an average equivalent pollution index of 15.37 (Fig 8).

The results of the cluster analysis are strongly corroborated by official water quality rankings. Provincial surface water environmental quality data from Henan (2020–2023) show that cities classified as high-performing-namely Sanmenxia, Jiyuan, and Xinyang-consistently ranked among the top, whereas those identified as low-performing-namely Shangqiu, Kaifeng, and Puyang-occupied the bottom positions, demonstrating clear agreement between the two assessments. Furthermore, the water environment index grading system developed by the Institute of Public and Environmental Affairs (IPE) classifies urban water quality into five tiers (excellent, good, average, poor, and very poor). The 2023 ratings for Henan Province were closely with our findings. Kaifeng, Puyang, and Shangqiu were categorized as poor, very poor, and poor, respectively. In contrast, Sanmenxia, Jiyuan, and Xinyang all received an excellent rating.

### Countermeasures for ANPSP

Targeted prevention and control measures, including source reduction, process control, and end-of-pipe treatment, should be developed for the different sources of agricultural non-point pollution and associated risk control zones.

It is essential to continuously reinforce efforts to effectively enhance public awareness of ANPSP prevention and mitigation. This can be conducted by encouraging active participation in reducing pollutants from rural household waste. Furthermore, it is crucial to promote the application of soil testing-based fertilization technology, as well as intelligent water and fertilizer control applications, to reduce the discharge of pollutants from fertilizers and improve the utilization efficiency of nitrogen, phosphorus, and compound fertilizers. Organic fertilizers can also be used to further enhance soil fertility. Government departments need to comprehensively consider multiple factors to reduce pollutant loads from livestock and poultry waste, such as environmental carrying capacity, resource conditions, market demands, and reasonable livestock and poultry farming. They should also clearly define the functional positioning and development direction of these areas. The management and direction of the aquaculture sector must be strengthened by formulating scientific and reasonable breeding plans, optimizing the structure of breeding varieties, and promoting breeding methods suitable for the local ecological environment. On the other hand, pollutant loads from farmland straw can be mitigated by ensuring effective management of solid waste classification, adopting treatment measures for different types of solid waste pollutants, and improving the treatment efficiency and resource utilization rate. For example, straw can be used as fertilizer and biomass energy.

The government should promote strict environmental supervision measures, particularly in the high-risk zones, including strict emission standards for livestock/poultry farming and aquaculture, a management system for agricultural inputs, and guidelines for agricultural solid waste treatment. For the medium-risk areas, a comprehensive monitoring and early warning system for ANPSP should be established to promptly identify and address pollution issues. Whereas in the low-risk areas, extensive environmental protection and education activities should be carried out to continuously enhance the public’s awareness against ANPSP.

## Discussion

Over the past two decades, the total pollution load in Henan Province exhibited an increasing-decreasing trend. Specifically, the 2001–2006 period exhibited an increasing trend, while a sharp reduction occurred in 2007, which might be due to the global financial crisis as a result of the impacts of rural economies and livestock production [41]. For instance, livestock and sheep populations in Henan Province decreased by 30.58 and 54.96%, respectively, compared with those in 2006. Subsequent fluctuations were observed, highlighting an increase in pollution from 2007 to 2013, followed by a pronounced decline from 2014 to 2019. These patterns were, in fact, consistent with those revealed by Ding et al. (2021) [25] and Ge et al. (2022) [19]. The recent downward trend reflects enhanced governance of NPSP in Henan, particularly through policy interventions. Notably, the national Zero Growth Policy for Chemical Fertilizers project implemented in 2015 resulted in continuous reductions in fertilizer and agricultural plastic film usage over five consecutive years, accompanied by increased grain yields. This underscores balanced and sustainable agricultural productivity in Henan Province through improved land management and technological adoption.

Rural households and livestock were the main sources of COD and TP emissions, respectively, which were similar with that in Shandong Province, Hubei Province, and Beijing City. Despite governmental incentives to revive agricultural activities after 2007, livestock breeding has remained limited due to the implementation of strict regulations on small-scale farming, as well as to urbanization-driven rural population decline, and low profitability in animal husbandry. Concurrently, improved rural waste management and farmer education have reduced household contributions to NPSP. Since 2004, various policies and initiatives related to energy efficiency have been implemented [42]. Electricity-conserving behavior of residents including households’ willingness to save electricity and their behavioral patterns in such savings can have a impact on emission reduction. Rural households with a healthy and old household head reduce the share of coal consumption, and household labors with an off-farm job and high level of education, and a large household size increase the share of liquefied petroleum gas (LPG) and electricity consumption [43]. The good economic condition of rural households contributes to the reduction of biomass consumption.

The changes in pollution sources since 2007 and higher application rates of chemical fertilizers were inconsistent with those revealed in a previous study in Guangdong Province, where livestock-dominated pollution loads [19]. In addition, national-scale analyses highlighted substantial livestock and fertilizers-associated pollution loads [27]. This anomaly might be attributed to the high fertilizer application in Henan Province, combined with reduced livestock numbers and enhanced manure treatment in specialized farms. Additionally, low phosphorus utilization efficiency (<30%) and severe leaching losses in saturated farmland soils [40] can exacerbate fertilizer-derived pollution loads. While national pollution census data highlight a contribution rate of the livestock sector to agricultural pollution at 54.46%. The unique agricultural structure and management practices in Henan Province have inverted this hierarchy.

Spatially, the COD, TN, and TP discharge loads exhibited decreased from the southeastern to the northwestern parts, which is consistent with the results of Li et al. [44]. The Huang-Huai-Hai Plain, a primary grain-producing region with flat topography and limited awareness among farmers of pollution risks, had disproportionately high emissions. Agricultural intensification, including frequent tillage, intercropping, and excessive agrochemical use, resulted in soil erosion, thereby promoting pollutant discharges. In contrast, forest areas in the western part of Henan exhibited lower pollutant loads due to reduced exogenous phosphorus inputs, enhanced rainfall interception by canopies [45], and improved soil infiltration capacity [1]. Forests and grasslands can effectively provide vital ecosystem services, thereby mitigating pollution discharges [46]. Despite progress, continuous spatial heterogeneity in pollutant loads was observed due to the historical overexploitation of central plains and challenges in farmer education. Achieving uniform pollution control requires stricter enforcement of existing policies and continued investment in farmer training. Future studies should, therefore, focus on the quantification of trade-offs between yield optimization and environmental sustainability under evolving climatic and socioeconomic conditions.

The spatiotemporal evolution of ANPSP was influenced by multiple factors, such as natural geographic conditions, agricultural economic structure, and national policies. In fact, the southern flat part of the Huang-Huai Plain and the dense river networks promoted pollutant diffusion. These effects are further enhanced by high cropping indices and excessive fertilizer inputs. Grain production increased by 60.7% after 2000. However, grain crops promoted TN and TP loads. Indeed, over 85% of these loads were attributed to rice paddies in the southern part of the study area. On the other hand, the spatial redistribution of livestock activities exacerbated COD emissions. The implementation of the Water Pollution Prevention Action Plan in 2015 reduced COD loads by 12% in key basins. Strategic investments in interception ditches decreased TP loads by 15–20% in Zhoukou/Shangqiu.

Zones with non-point source pollution control were clustered into three classes. These classes were, in fact, influenced by national policies, natural ecology, and economic structure [47]. In Zone I, the rate of large-scale livestock and poultry breeding in cities, such as Anyang and Xinxiang, increased to 78% in 2020. These activities were governed by the implementation of policies, namely the Yellow River Basin Ecological Protection and High-Quality Development Plan. In Zone III (Xinyang), the proportion of cultivated land in the Dabie Mountain, with a slope greater than 15°, reached 64%. In this area, the development of intensive agriculture is restricted. The input of chemical fertilizers per unit area (320 kg/ha) accounted for only 51.6% of that in the Zone I area, demonstrating the natural control effect of topographic factors on the pollution loads. The proportion of agricultural output value in the suburbs of Zhengzhou increased from 12% in 2001 to 29% in 2023. Nevertheless, due to the fragmentation of cultivated land, the marginal cost associated with non-point source pollution control increased by 37%, resulting in high output value with high pollution loads.

## Conclusions

(1) The total ANPSP loads showed a continuous decrease by 25.24% in Henan Province from 2001 to 2023. Rural household waste and livestock were the main sources of the COD and TP emissions. Livestock and farmland straw were the main sources of TN in the 2001–2016 and 2017–2023 periods, respectively.
(2) The highest total ANPSP loads were found in the southern and eastern parts of Henan Province, while the lowest loads were observed in the northern and western parts. Nanyang, Zhoukou, Zhumadian, Shangqiu, and Xinyang were the cities where the highest ANPSP and equivalent pollution loads were found over the 2001–2023 period.
(3) The decoupling state of ANPSP emissions and agricultural output values in Henan Province over the 2001–2023 period showed a fluctuating trend, mainly consisting of strong and weak decoupling.
(4) The cities with the high-risk class included Kaifeng, Puyang, and Shangqiu, the medium-risk areas included Pingdingshan, Jiaozuo, Zhumadian, Luoyang, Xinxiang, Xuchang, Zhoukou, Anyang, Luohe, Zhengzhou, Nanyang, and Hebi, and the low-risk areas were Sanmenxia, Xinyang, and Jiyuan.
(5) This study quantifies the effects of multiple driving factors on ANPSP changes, including grain production capacity, population, and pollution intensity. Moreover, the study elucidates the regional disparities in the contribution ratios of these factors and their dynamic evolution patterns. These findings not only provide strong support for local governments in formulating strategies for controlling ANPSP but also lay a foundation for future research in similar regions.

## Supporting information

S1 File **S1 Appendix** Spatio-temporal patterns of the equal standard emission of COD in four typical stages in Henan Province, China. **S2 Appendix** Spatiotemporal patterns of the equal standard emission of TN in four typical stages in Henan Province, China. **S3 Appendix** Spatiotemporal patterns of the equal standard emission of TP in four typical stages in Henan Province, China. **S4 Appendix** The mean values of total emission in Henan Province during 2001 and 2023. **S5 Appendix** The mean values of total emission intensity in Henan Province during 2001 and 2023. **S6 Appendix** The percent pollution values of different agricultural non-point sources pollutants (TN、TP and COD) between 2001 and 2023 in Henan Province, China.
(7Z)

## Abbreviation:

ANPSP: Agricultural Non-point Source Pollution
COD: Chemical oxygen demand
TN: Total nitrogen
TP: Total phosphorous
NPS: Non-point Source Pollution
SWAT: Soil and Water Assessment Tool
AGNPS: Agricultural Non-point Source Model
ANSWERS: Areal Nonpoint Source Watershed Environment Response Simulation
AFAF: Agriculture, Forestry, Animal husbandry and Fishery
OECD: Organization for Economic Cooperation and Development
EU: Elementary Unit
ESPL: Equal Standard Pollution Load
EPI: Equal Pollution Index
SPSS: Statistical Program for Social Sciences.
